# Discrepancies in Splenic Size Measurement: A Comparative Analysis of Ultrasound and Computed Tomography

**DOI:** 10.3390/diagnostics14080789

**Published:** 2024-04-10

**Authors:** Hun Woo Lee, Hee Sun Park, Sungeun Park, Mi Hye Yu, Young Jun Kim, Sung Il Jung

**Affiliations:** 1 Department of Radiology, Konkuk University Medical Center, 120-1, Neungdong-ro, Gwangjin-gu, Seoul 05030, Republic of Korea; 20200120@kuh.ac.kr (H.W.L.); 20210126@kuh.ac.kr (S.P.); 20140130@kuh.ac.kr (M.H.Y.); yjkim@kuh.ac.kr (Y.J.K.); radsijung@kuh.ac.kr (S.I.J.); 2 Department of Radiology, Konkuk University School of Medicine, 120-1, Neungdong-ro, Gwangjin-gu, Seoul 05030, Republic of Korea

**Keywords:** spleen size, measurement discrepancy, ultrasound, computed tomography

## Abstract

The accurate measurement of splenic size is essential for the diagnosis and management of various gastrointestinal and hematological conditions. While ultrasound (US) and computed tomography (CT) are widely used imaging modalities for assessing splenic size, discrepancies between their measurements have been observed in clinical practice. This study aimed to analyze the measurement differences between US and CT and identify factors influencing these differences. A retrospective analysis of 598 asymptomatic patients who underwent both abdominal US and CT was conducted. Measurements of splenic size obtained from US, axial CT, and coronal CT scans were compared, and various factors such as patient demographics, operator experience, and imaging parameters were evaluated to elucidate their impact on the measurement discrepancies. The results revealed that US consistently underestimated splenic size compared to CT. The magnitude of the discrepancy was influenced by factors such as patient age, body mass index (BMI), depth of the spleen from skin on US and that on CT, visibility of the splenic hilum on US, sonic window quality, and operator experience. This study underscores the importance of considering these factors when interpreting splenic measurements obtained from different imaging modalities in clinical practice.

## 1. Introduction

The spleen is the largest organ of the reticuloendothelial system [1]. Evaluation of splenic size plays a crucial role in the diagnosis and management of various medical conditions, including liver disease, immune disorders, and hematological malignancies [1,2,3]. Furthermore, accurate measurement of splenic size is indispensable for monitoring disease progression, assessing treatment response, and predicting clinical outcomes [4,5].

Various imaging modalities are employed for assessing splenic size, including ultrasound (US) [6,7,8,9,10,11], computed tomography (CT) [12,13,14,15,16,17,18,19], magnetic resonance imaging (MRI) [20,21], and nuclear scintigraphy [22,23,24], among which US and CT hold prominence [25,26]. US is often the first imaging method for evaluation of splenic size due to its widespread availability, cost effectiveness, and lack of ionizing radiation [2]. However, assessment of spleen size using US is sometimes challenging because the spleen is located in the intercostal space and shadowing from the ribs, bowel gas, and overlying lungs is frequent. In addition, the visibility of the spleen is dependent on respiration status, and the shape and position of the spleen are quite variable [27]. Spleen size is also affected by various parameters, such as age, sex, height, weight, body mass index (BMI), and scanning position [1,27]. By contrast, CT offers superior spatial resolution and multiplanar imaging capabilities, making it a reliable and accurate imaging modality. CT is considered the gold standard for splenic volume measurements, but it exposes the patient to ionizing radiation [25,26].

Despite the widespread use of these modalities, discrepancies in splenic size measurements between US and CT have been observed in clinical practice, posing challenges for accurate interpretation and clinical decision making. Previous studies have compared splenic size measurements obtained from US and CT imaging and have reported variable degrees of correlation between the two modalities [1,25]. These inconsistencies underscore the need for further investigation into the factors influencing the measurement differences between US and CT. Consequently, there is a critical need to elucidate the factors influencing measurement discrepancies between US and CT to enhance the accuracy and reliability of splenic size measurements in clinical practice.

Therefore, the primary objective of this study was to assess the discrepancies in splenic size determined by US and CT in a screening population and to elucidate the factors that affect the discrepancies between the two imaging modalities. By identifying these factors, we aim to provide insight that can enhance the accuracy and reliability of splenic size measurements in clinical practice.

## 2. Materials and Methods

### 2.1. Study Population and Data Collection

Our institutional review board approved this retrospective study, which waived the requirement for written informed consent due to the retrospective study design. Between January 2021 and June 2021, 11,402 asymptomatic adult patients underwent abdominal US at our institution’s healthcare center as a routine health screening. Of these patients, 718 (6.3%) who underwent abdominal CT on the same day for further evaluation of the liver, gallbladder, pancreas, etc., were included. Among them, 120 were excluded because of the absence of splenic US images.

**Figure 1:**
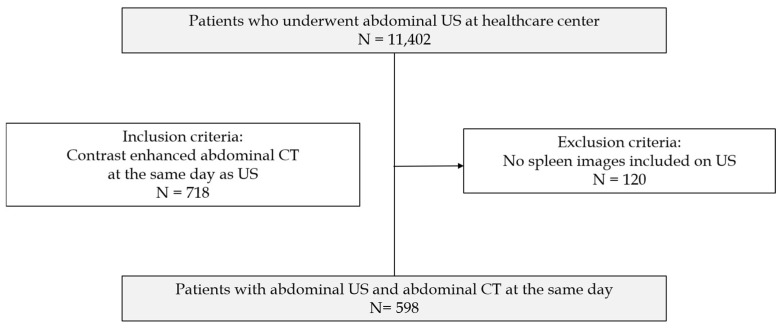
Flow chart of the patient population and study design.

The final study cohort comprised 598 patients, including 316 men and 282 women, with a mean age of 53.1 years (range: 25–82 years). A detailed flow chart outlining the patient selection process is provided in Figure 1.

### 2.2. Ultrasound (US) Examinations

All patients were instructed to fast for at least 8 h before the US exam. Six board-certified radiologists with at least 5 years of experience in abdominal sonography performed the US examinations. The spleen was imaged on a standard grayscale using a 2–5 MHz curvilinear transducer (iU22; Philips Healthcare, Bothell, WA, USA). During scanning, the operating radiologist determined the scan position (supine or right lateral decubitus) and respiration control.

### 2.3. Computed Tomography (CT) Examinations

Computed tomography (CT) was performed using a 64-channel scanner (Philips Ingenuity, Philips Healthcare, Cleveland, OH, USA). The CT scanner was set to the following parameters: detector collimation, 64 × 0.625 mm; spiral pitch, 0.798; tube voltage, 120 kVp; and tube current, 142–400 mAs with automatic exposure control (iDose; Philips Healthcare). Images were reconstructed at a section thickness of 3 mm and an interval of 3 mm. Triple-phase dynamic CT or single-portal phase CT were used. Unenhanced scans were obtained, followed by arterial-, portal-, and delayed-phase scans, after an intravenous injection of 150 mL of iopromide (Ultravist 370; Bayer Schering Pharma, Berlin, Germany) administered at a rate of 3 mL/s with an autonomic injector. Coronal reformatted images were created using the source CT dataset, with the slice thickness and reconstruction interval set to 3 mm.

### 2.4. Image Analysis

**Figure 2:**
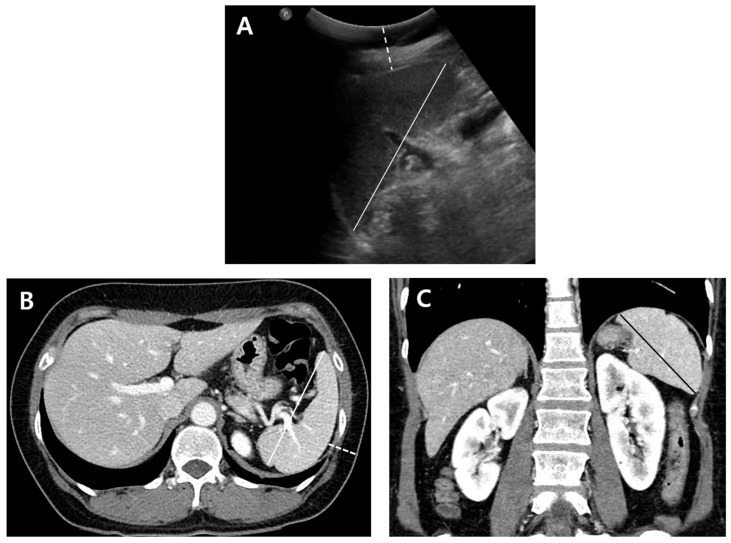
Measurement of spleen on US and CT in a 54-year-old woman. (**A**) Longitudinal diameter of the spleen in supine position (solid line) and the depth of the spleen from the skin (dotted line) are measured on US. (**B**) The longest diameter of the spleen on axial CT (solid line) and the distance of the spleen from the skin (dotted line) are measured. (**C**) The longest diameter of the spleen on coronal CT (solid black line) is measured. Splenic lengths measured on US (**A**), axial CT (**B**), and coronal CT (**C**) were 8.7 cm, 10.5 cm, and 8.9 cm, respectively. Depths of the spleen from the skin on US (**A**) and CT (**B**) were 2.1 cm and 2.3 cm, respectively.

All US and CT images were transferred and loaded onto a picture archive and communication system (Centricity PACS 6.0 SP9; GE Healthcare, Waukesha, WI, USA) for interpretation. Two board-certified radiologists with 15 and 5 years of experience in abdominal radiology, respectively, who did not perform the US examination, reviewed the US images in consensus. The observers measured the longitudinal diameter of the scanned spleen and the shortest distance from the skin to the spleen using electronic calipers (Figure 2). The observers rated the visibility of the splenic hilum and sonic window on a three-point scale (0 = poor, 1 = moderate, and 2 = good).

One observer (less experienced) measured the longest splenic diameter on axial and coronal contrast-enhanced CT scans. The distance from the skin in the left flank area, where the US probe was presumed to be placed, to the splenic capsule was measured (Figure 2). The review sessions of the US and CT image sets had a time interval of at least two weeks to minimize recall bias. The splenic diameter differences between US and axial CT scans (CT_ax_-US) and US and coronal CT scans (CT_cor_-US) were calculated. Since the CT scan measurement followed a standard procedure that is more reliable, CT measurement was used as a gold standard in this study.

### 2.5. Evaluation of Factors That Affect the Spleen Measurement Discrepancies

Clinical and imaging parameters affecting the discrepancies in splenic size measurement between US and CT included patient sex (male or female), age (<70 vs. ≥70), BMI (weight [kg]/height [m^2^]; underweight or normal ranges < 25 vs. overweight ≥ 25), depth of the spleen from the skin on US (<3 cm vs. ≥3 cm), depth of the spleen from the skin on CT (<3 cm or ≥3 cm), visibility of splenic hilum on US (grades 0, 1, and 2), sonic window quality of the spleen (grades 0, 1, and 2), and US operator (*n* = 6).

### 2.6. Statistical Analysis

The Shapiro–Wilk test was used to determine whether continuous variables followed a normal distribution. The agreement between US and CT measurements of splenic size was assessed using intraclass correlation coefficients (ICCs) with 95% confidence intervals (CIs). ICC values greater than 0.75 were considered indicative of excellent agreement, while values between 0.40 and 0.75 were considered indicative of moderate agreement, and values less than 0.40 were considered indicative of poor agreement. Correlations between the measured splenic diameters on US, CT_ax,_ and CT_cor_ were determined using Pearson’s correlation. The spleen diameters measured on US and CT were compared using a paired *t*-test. Comparisons of absolute values of diameter difference (|CT_ax_-US| and |CT_cor_-US|) between groups according to patient sex, age, BMI, and depth of the spleen from the skin on US or CT were performed using an independent *t*-test. Comparisons of |CT_ax_-US| and |CT_cor_-US| between groups according to the US operator, splenic hilum visibility, and the spleen’s sonic window were performed using repeated measures analysis of variance. Statistical significance was set at *p* < 0.05. Statistical analyses were performed using commercially available software (IBM^®^ SPSS^®^ Statistics, version 28.0.0.0; MedCalc^®^, version 22.017).

## 3. Results

### 3.1. Comparison and Correlation of Spleen Diameter Measurements between US and CT

**Figure 3:**
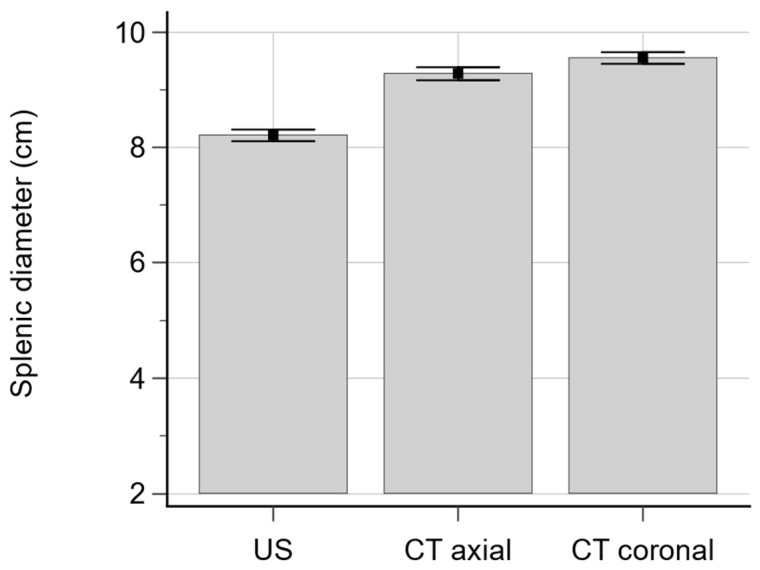
Bar graphs showing splenic length measured on US and CT. Splenic diameter on US, CT_ax_, and CT_cor_ was 8.21 cm ± 1.32 (mean ± standard deviation), 9.29 cm ± 1.44, and 9.56 cm ± 1.23, respectively.

Continuous variables followed a normal distribution (*p* > 0.05, Shapiro–Wilk test). Splenic diameter measured on US, CT_ax_, and CT_cor_ was 8.21 cm ± 1.32 (mean ± standard deviation), 9.29 cm ± 1.44, and 9.56 cm ± 1.23, respectively (Figure 3). The diameter significantly differed between US and CT_ax_ and US and CT_cor_ (*p* < 0.001). As for diameter difference, CT_ax_-US was 1.08 ± 1.25 and CT_cor_-US was 1.35 ± 1.02, which suggested that US underestimated spleen diameter compared with CT_ax_ and CT_cor_. The percentage differences were 16.93% and 16.58% for US and CT_ax_ and US and CT_cor_, respectively.

**Figure 4:**
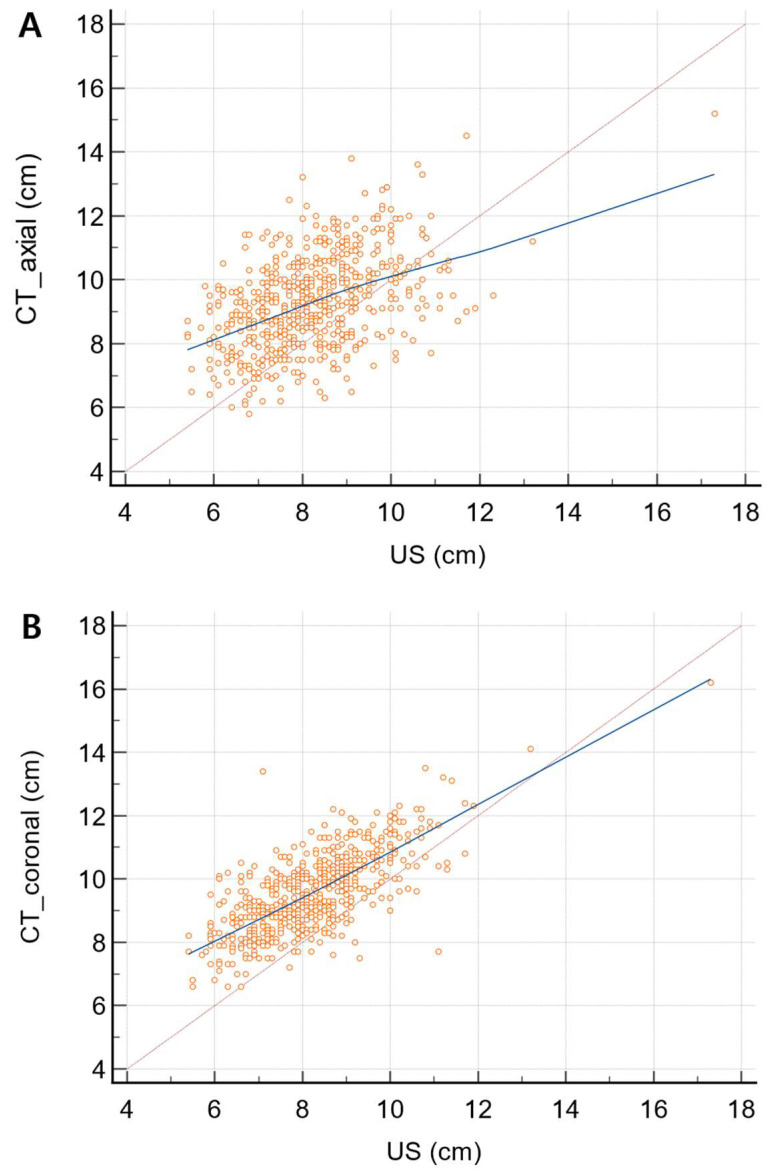
Scatterplots of spleen diameter measured on CT_ax_ (**A**) and CT_cor_ (**B**) as a function of that measured on US. Solid lines represent best-fit linear regression, and dotted lines represent the line of equality. Spleen diameter showed a moderate positive correlation between US and CT_ax_ (Pearson’s correlation coefficient r = 0.454), while it showed a strong positive correlation between US and CT_cor_ (r = 0.72).

The agreement between US and CT_ax_ was moderate, with an ICC of 0.454 (95% CI of 0.386–0.514) (*p* < 0.0001), and that between US and CT_cor_ was excellent, with an ICC of 0.718 (95% CI of 0.677–0.755) (*p* < 0.0001). Spleen diameter measurements showed a strong positive correlation between US and CT_cor_ (Pearson’s correlation coefficient r = 0.72) and a moderately positive correlation between US and CT_ax_ (r = 0.454), with statistical significance (*p* < 0.0001) (Figure 4).

### 3.2. Association of Measurement Discrepancies with Clinical and Imaging Parameters

Table 1 displays the spleen size discrepancies between US and CT according to the clinical and imaging parameters. The older age group (*n* = 47) showed a bigger |CT_ax_-US| (1.72 ± 1.29) than the younger age group (*n* = 551, 1.47 ± 1.00) (*p* = 0.01) and a bigger |CT_cor_-US| (1.54 ± 0.96) than younger age group (1.43 ± 0.87) (*p* = 0.302). The high BMI group (*n* = 197) showed a bigger |CT_ax_-US| (1.65 ± 1.06) than the normal BMI group (*n* = 399, 1.40 ± 1.00) (*p* = 0.022) and a bigger |CT_cor_-US| (1.53 ± 0.99) than the normal BMI group (1.40 ± 0.81) (*p* = 0.135).

The group with depth of spleen from skin measured on US ≥ 3 cm (*n* = 81) showed a bigger |CT_cor_-US| (1.76 ± 1.1) than the group with depth of spleen from skin on US < 3 cm (*n* = 517, 1.4 ± 0.83) (*p* = 0.04) and a bigger |CT_ax_-US| (1.77 ± 1.07) than the group with depth of spleen from skin on US < 3 cm (1.44 ± 1.01) (*p* = 0.719). Similarly, the group with depth of spleen from skin measured on CT ≥ 3 cm (*n* = 103) showed a bigger |CT_cor_-US| (1.54 ± 1.15) than the group with depth of spleen from skin on CT < 3 cm (*n* = 495, 1.43 ± 0.81) (*p* = 0.016) and a bigger |CT_ax_-US| (1.51 ± 0.97) than the group with depth of spleen from skin on CT < 3 cm (1.48 ± 1.03) (*p* = 0.599).

As for visibility of the splenic hilum on US, |CT_ax_-US| was 1.54 ± 1.07 in the poor visibility group, 1.18 ± 0.81 in the moderate visibility group, and 1.15 ± 0.9 in the good visibility group (*p* = 0.006). In addition, |CT_cor_-US| was 1.54 ± 0.9 in the poor visibility group, 1.22 ± 0.78 in the moderate visibility group, and 1.13 ± 0.7 in the good visibility group (*p* < 0.001). As for the sonic window quality of the spleen, |CT_cor_-US| was 1.61 ± 0.95 in the poor sonic window group, 1.37 ± 0.73 in the moderate sonic window group, and 1.19 ± 1.03 in the good sonic window group, and the difference was statistically significant (*p* < 0.001). But |CT_ax_-US| was 1.61 ± 0.95 in the poor sonic window group, 1.37 ± 0.73 in the moderate sonic window group, and 1.40 ± 0.96 in the good sonic window group, and the difference was not significant (*p* = 0.14).

|CT_cor_-US| of the six US operators was 1.57 ± 0.86, 1.63 ± 0.78, 1.54 ± 0.85, 1.28 ± 0.87, 1.37 ± 0.96, and 1.38 ± 0.81, respectively, and it differed significantly among the operators (*p* = 0.035). |CT_ax_-US| of the six operators was 1.64 ± 1.04, 1.36 ± 0.91, 1.46 ± 0.93, 1.49 ± 0.98, 1.49 ± 1.16, and 1.39 ± 1.11, respectively (*p* = 0.64) (Figure 3).

## 4. Discussion

Our results demonstrated that US measurement of splenic length showed a moderate to strong correlation with splenic length measured on CT. Previous studies regarding splenic size measurement have reported a good correlation between splenic length on US and splenic volume calculated from CT or between splenic volume measured on US and CT [1,25]. Our results are in accordance with these studies, although we simply measured the maximum length of the spleen on both US and CT and did not use splenic volume calculations.

However, in our study, the splenic size measurements by US and CT were significantly different. The mean differences were 1.08 ± 1.25 (mean ± standard deviation) in CT_ax_-US and 1.35 ± 1.02 in CT_cor_-US, and the percentage differences were 16.93% and 16.58%, respectively. The shape and position of the spleen are individually variable [27], and various methods of spleen measurement have been used in the literature. On US, the measurement planes are coronal [9,28,29], sagittal [30,31], transverse [25,30,31,32], and parallel to the intercostal space [33]. The types of measurements include maximum length [9,25,28,29,30,31,32,33], craniocaudal length [25], width [9,28,29,30,31,32,33], depth [32], thickness [30], and anteroposterior dimension [33]. In routine clinical practice, the majority of US operators perform spleen measurements on longitudinal scans only [10,14]. On CT, the spleen is measured in the transverse or coronal plane [1,34].

In our study, six US operators scanned the spleen and measured the splenic size in their own way; however, they scanned the spleen where the maximum length of the spleen could be measured, which was the oblique coronal plane or sagittal plane in most cases. By contrast, spleen measurements on CT were performed in either the axial or the coronal plane. Therefore, there was a difference in the scan plane between US and CT in our study, which may have led to a size difference between the two modalities.

Our study results revealed that US significantly underestimated splenic size compared with CT. We investigated the possible effects of patient age, BMI, depth of the spleen from the skin, splenic hilar visibility on US, the sonic window quality of the spleen, and different US operators, which could affect the confidence of US measurement. According to our results, the older age group (equal to or older than 70 years) showed a significantly larger size difference between CT_ax_ and US than the younger age group. The spleen is located in the left hypochondrium, where the inferior thoracic rib cage entirely covers it, and US scanning of the spleen usually requires full inspiration so that the diaphragm moves downward [35]. Since it is relatively difficult for elderly patients to control respiration, this could interfere with the US measurement of maximum spleen length.

Patients with a high BMI showed larger size differences between the CT_ax_ and US groups than the normal or low BMI groups. Overweight patients usually have larger volumes of visceral and subcutaneous fat, frequently making the entire spleen length measurement on US difficult, leading to an underestimation of spleen length. Likewise, the depth of the spleen from the skin measured on US and CT can influence the measurement accuracy of the splenic length on US for similar reasons. In our study, a depth of the spleen from the skin > 3 cm showed a significantly larger size difference between CT_cor_ and US.

According to our results, the degree of splenic hilar visibility on US and sonic window quality significantly affected the size discrepancies between US and CT. Maximum splenic length was obtained when the splenic hilum was visualized along the longitudinal axis. However, in our study, good or moderate splenic hilar visibility was observed in 159 patients (26.6%), whereas poor hilar visibility was observed in 439 patients (73.4%). There are many reasons for poor splenic hilar visibility on US, including anatomic factors such as ribs, bowel gas, or lung shadow [27]; patient factors such as incomplete inspiration, obesity, or experience of US operators; etc. Along with visibility of the splenic hilum, the quality of the sonic window, defined as the clarity and depth of penetration of the ultrasound beam, significantly influenced the measurement accuracy of US. Suboptimal sonic window quality was associated with larger measurement discrepancies between US and CT. These findings emphasize the importance of optimizing imaging parameters and operator technique to enhance measurement consistency.

US was performed by six radiologists in our study, and the splenic size discrepancies between CT_cor_ and US significantly differed between the operators. Since this study was performed retrospectively, operators performed US with the scan plane and scan position in their own way, which may have affected splenic size measurement and different US experiences depending on each operator. Lamb et al. reported that the splenic length measured on a longitudinal section with the patient in the right lateral decubitus position was closely correlated with the splenic volume measured using helical CT (r = 0.86) [1]. In this study, measurements performed in the right lateral decubitus position showed a stronger correlation with CT volume than those performed in the supine position. Standardization of imaging protocols and ongoing operator training are essential for minimizing measurement discrepancies and ensure reliable interpretation of splenic measurements in clinical practice.

Despite the valuable insight gained from this study, several limitations should be acknowledged. First, we only measured one dimension of the spleen: the maximum length in the oblique coronal or sagittal plane on US and that in axial and coronal CT scans. Previous studies used various parameters in spleen measurement, including length, width, and depth, and performed volume measurement using three-dimensional parameters [1,2,25,34] because spleens have variable shapes, and single-length measurements frequently do not reflect the real spleen size. However, several reports have correlated linear US measurements with CT volume [1,34]. Spleen volume measurement in every patient is a time-consuming task in the clinical routine, and this study aimed to determine the discrepancies between US and CT in the conditions under which daily practice is performed. Second, this is a retrospective study and only one measurement per US study was taken; thus, the repeatability of the measurements cannot be assessed. Third, the patient population consisted of individuals undergoing routine health screenings from a single institution, which might limit the generalizability of the findings to a broader population and potentially introduce selection bias. The exclusion of patients with specific medical conditions, such as gastrointestinal and hematological diseases, might affect the applicability of the results to certain clinical scenarios. Indeed, the portion of patients with splenomegaly was very small. Mean spleen length was 9.3 cm and 9.7 cm on CT_ax_ and CT_cor_, respectively, and the number of patients with splenomegaly (spleen size > 12 cm) was only *n* = 5 and 7, respectively. Because enlarged spleens are more easily seen on US than normal-sized spleens, including patients with splenomegaly would have produced different results. In addition, the sample size (*n* = 598) may not be sufficient to draw definitive conclusions, especially considering the potential variability in splenic size within the population. Finally, the study did not assess the clinical outcomes or impact of the observed discrepancies on patient management or prognosis. While accurate measurement of splenic size is essential for diagnosis and treatment planning, the clinical significance of minor discrepancies between US and CT measurements remains unclear. Further research is needed to evaluate the clinical implications of these discrepancies and their impact on patient care.

Despite these limitations, this study provides valuable insight into the discrepancies between US and CT measurements of splenic size and highlights the importance of standardized imaging protocols, operator training, and quality assurance measures in clinical practice. Clinicians should be aware of the inherent limitations of US imaging, such as poor visualization of deep-seated structures and technical challenges related to bowel gas interference, which may lead to underestimation of splenic size. In cases where accurate measurement of splenic size is critical for clinical decision making, CT may be considered as a complementary imaging modality to confirm US findings and provide more reliable measurements.

Future research should focus on validating the findings of this study in larger multicenter cohorts with diverse patient populations. Prospective studies are needed to assess the impact of different imaging techniques and operator experience on the measurement accuracy of splenic size by US and CT. Additionally, studies evaluating the clinical implications of splenic size measurement discrepancies on patient outcomes and management strategies are warranted.

## 5. Conclusions

In conclusion, this study highlights the measurement discrepancies between US and CT in assessing splenic size and identifies factors influencing these discrepancies. US consistently underestimated splenic size compared to CT, and patient demographics including patient age and BMI, depth of the spleen from the skin measured on US and CT, visibility of the splenic hilum on US, sonic window quality, and operator experience were found to affect the accuracy of splenic size measurement by US. Clinicians should consider these factors when interpreting splenic measurements obtained from different imaging modalities in clinical practice. Further research is warranted to validate these findings and optimize imaging techniques for accurate assessment of splenic size.

## Figures and Tables

**Table 1:** Spleen size discrepancies between US and CT according to the clinical and imaging parameters.

Parameter		N	|CT_ax_-US|	*p*-Value	|CT_cor_-US|	*p*-Value
Age	≥70	47	1.72 ± 1.29	0.01 *	1.54 ± 0.96	0.302 *
	<70	551	1.47 ± 1.00		1.43 ± 0.87	
BMI	≥25	197	1.65 ± 1.06	0.022 *	1.53 ± 0.99	0.135 *
	<25	399	1.40 ± 1.00		1.40 ± 0.81	
Depth of spleen from skin on US	≥3 cm	81	1.77 ± 1.07	0.719 *	1.76 ± 1.1	0.04 *
	<3 cm	517	1.44 ± 1.01		1.4 ± 0.83	
Depth of spleen from skin on CT	≥3 cm	103	1.51 ± 0.97	0.599 *	1.54 ± 1.15	0.016 *
	<3 cm	495	1.48 ± 1.03		1.43 ± 0.81	
Visibility of splenic hilum on US ^1^	0	439	1.54 ± 1.07	0.006 ^†^	1.54 ± 0.9	<0.001 ^†^
	1	102	1.18 ± 0.81		1.22 ± 0.78	
	2	57	1.15 ± 0.9		1.13 ± 0.7	
Sonic window of spleen ^2^	0	245	1.58 ± 1.09	0.14 ^†^	1.61 ± 0.95	<0.001 ^†^
	1	282	1.47 ± 1.01		1.37 ± 0.73	
	2	71	1.40 ± 0.96		1.19 ± 1.03	
US operator	1	76	1.64 ± 1.04	0.64 ^†^	1.57 ± 0.86	0.035 ^†^
	2	74	1.36 ± 0.91		1.63 ± 0.78	
	3	111	1.46 ± 0.93		1.54 ± 0.85	
	4	134	1.49 ± 0.98		1.28 ± 0.87	
	5	132	1.49 ± 1.16		1.37 ± 0.96	
	6	50	1.39 ± 1.11		1.38 ± 0.81	

Note—Data are mean ± standard deviation; N = number; BMI = body mass index; * Independent *t*-test; ^†^ Analysis of variance; ^1,2^ Degree = 0 (poor), 1 (moderate), 2 (good).

## Data Availability

Data are contained within the article.

## Abbreviations

US	ultrasound
CT	computed tomography
BMI	body mass index
MRI	magnetic resonance imaging
CT_ax_	axial CT scan
CT_cor_	coronal CT scan
ICC	intraclass correlation coefficient
